# Precise partial root-zone irrigation technique and potassium-zinc fertigation management improve maize physio-biochemical responses, yield, and water use in arid climate

**DOI:** 10.1186/s12870-024-05467-w

**Published:** 2024-08-15

**Authors:** Ayman M. S. Elshamly, A. S. Abaza

**Affiliations:** 1 Water Studies and Research Complex. National Water Research Center, Cairo, Egypt; 2 National Water Research Center, Research Institute for Groundwater, El-Kanater, El-Khairiya, Egypt

**Keywords:** *Zea mays*, Nutrient uptake, Drought stress, Deficit irrigation

## Abstract

**Background:**

To optimize irrigation water use and productivity, understanding the interactions between plants, irrigation techniques, and fertilization practices is crucial. Therefore, the experiment aims to assess the effectiveness of two application methods of potassium humate combined with chelated zinc under partial root-zone drip irrigation techniques on maize nutrient uptake, yield, and irrigation water use efficiency across two irrigation levels.

**Methods:**

Open-field experiments were carried out in two summer seasons of 2021 and 2022 under alternate and fixed partial root-zone drip irrigation techniques to investigate their impacts at two irrigation levels and applied foliar and soil applications of potassium humate or chelated zinc in a sole and combinations on maize.

**Results:**

Deficit irrigation significantly increased hydrogen peroxide levels and decreased proline, antioxidant enzymes, carbohydrate, chlorophyll (a + b), and nutrient uptake in both partial root-zone techniques. The implementation of combined soil application of potassium humate and chelated zinc under drought conditions on maize led to varying impacts on antioxidant enzymes and nutritional status, depending on the type of partial root-zone technique. Meanwhile, the results showed that fixed partial root-zone irrigation diminished the negative effects of drought stress by enhancing phosphorus uptake (53.8%), potassium uptake (59.2%), proline (74.4%) and catalase (75%); compared to the control. These enhancements may contribute to improving the defense system of maize plants in such conditions. On the other hand, the same previous treatments under alternate partial root zone modified the defense mechanism of plants and improved the contents of peroxidase, superoxide dismutase, and the uptake of magnesium, zinc, and iron by 81.3%, 82.3%, 85.1%, 56.9%, and 80.2%, respectively.

**Conclusions:**

Adopting 75% of the irrigation requirements and treating maize plants with the soil application of 3 g l^−1^ potassium humate combined with 1.25 kg ha^−1^ chelated zinc under alternate partial root-zone technique, resulted in the maximum root length, leaf water content, chlorophyll content, yield, and irrigation water use efficiency.

## Introduction

Agricultural yield is affected by a range of challenges, including scarcity of water resources and drought stress [1, 2]. Hence, innovative practices and sustainable water management strategies are required especially in countries characterized by arid climates like Egypt [3, 4]. Egypt relies on the Nile River, which provides 55 billion cubic meters per year accounts for 98% of renewable water resources [5]. Irrigated agriculture consumes 89% of Nile water, and the rest goes to industrial and domestic uses [6]. However, there is a growing gap in the Egyptian water demand between total renewable water resources and actual water use, which was in 2020 about 21 billion cubic meters [7]. This gap is supposed to reach 51 billion cubic meters, only to cover the domestic crop production by 2050 [8]. Therefore, farmers in similar conditions would need to rely on the limited irrigation water strategies to produce food, its amount depends on stress intensity, plant resilience, technology, and skills.

In this respect, drought stress is one of the biggest yield limiting factors for many crops especially for maize (*Zea mays* L.) [9]. In Egypt, while the total cultivated area of maize reached 871,076 hectares produced 8.2 million tons in 2020; there is a large gap between production and consumption reached about 45% [10, 11]. Fisher et al. [12] indicated that 40% of Africa's maize-planting area is affected by occasional drought. Maize developing under drought stress conditions demonstrates specific physiological, molecular, biochemical, and morphological responses [13], including reduced cell turgor, water potential in plant tissues, and carbon dioxide (CO_2_) exchange [14], lower chlorophyll content and photosynthetic efficiency [15], which eventually resulting in a decrease in maize yield [16, 17].

Hence, it is vital to closely monitor plant water status and plant resilience under drought stress conditions [18]. One of the valuable indicators for evaluating plant drought tolerance is the leaf water content [19]. Other important indicators are the ability of plants to accumulate nutrients [20] and osmolytes according to drought intensity [21].

To combat drought stress, plants generally produce and store cellular osmolytes, antioxidants, and secondary metabolites to maintain water status, subcellular structures, and balance homeostasis [22]. Proline is an important compatible solute that performs the role of reactive oxygen species scavengers and stores carbon and nitrogen in stressed plants [23]. The crucial roles of antioxidants are to scavenge free radicals from plant tissues and help to prevent or decrease the damage caused by oxidation [24]. Consequently, investigating the mechanisms underlying the plant reactions under drought conditions may be the key for developing unique techniques to improve plant tolerance and secure global food demands [25].

While the previous productions of osmolytes, antioxidants, and secondary metabolites can improve plant resilience to drought stress, still the plants get affected. To ensure better growth conditions for plants, several techniques have been adapted to counteract the harmful effects of drought stress.

Among these techniques used to reduce the aforementioned negative influences caused by drought, especially in arid areas are regulated deficit irrigation (RDI) and partial root-zone drying (PRD). In this respect, PRD technique represents a modified form of deficit irrigation, as reported by [1, 26, 27]. In the RDI technique (called fixed partial root-zone irrigation), the irrigation water amounts are applied less than the crop’s water demands [28], causing stricter drought stress during a specific period of plant phenological stages [29, 30]. That returned several benefits such as yield increments and irrigation water saving [31–33]. Under PRD irrigation technique, only half of the plant's roots is irrigated during every irrigation time, allows one side of the root to absorb water and the other half to remain dry until the following irrigation time [34]. Accordingly, PRD involves growing half of the root in watered soil and placing the other portion in dry soil, is an essential irrigation technique [35]. Hence, in Turkey during the seasons of 2010 and 2011, Sahin et al. [36] reported that PRD irrigation technology reduced water consumption by 42% while biomass decreased by 24% compared to full irrigation. Additionally, PRD technique theoretically allows the presence of a permanent degree of stress that improves the physio-chemical traits of the plant including antioxidant enzymes, proline, and soluble sugars [37–39]. However, despite improved osmoprotectants, some crops grown under PRD generally exhibited lower leaf water content, polyphenols, chlorophylls, and yield [37, 38] and higher production of ROS species [38]; possibly making this technique PRD acceptable in areas affected by limited water resources by improving these aforementioned negative influences [40]. Hence, it would be effective, especially supplying stressed maize plants with exogenous applications which can play a significant role in enhancing plants' drought resistance under both irrigation techniques (RDI and PRD).

In this respect, previous studies have shown the effective role of potassium and zinc (Zn) applications in reducing the harmful effects of water stress on crop productivity and irrigation water use efficiency [21, 41–43]. Potassium acts an essential role in decreasing abiotic stress on plants such as extreme cold, water deficit, and bright light [44]. Potassium humate (Kh) produced from brown charcoal lignite, which is naturally aromatic and rich in carboxylic and phenolic groups, enhances biological activity, increases pH buffering, improves the physical structure of the soil, and hastens the plant's uptake of nutrients [45]. On the other side, Zn is considered one of the required essential micronutrients for plant growth and development. Although the normal concentration of Zn in plant tissues is about 30–100 mg kg^−1^, Zn plays a crucial role in more than 300 enzymes [46–48]. Therefore, numerous studies have demonstrated the advantages of Zn applications on improving water relations within plants (leaf water potential and stomatal conductance), plant cell membrane stability, chlorophyll formation, osmolyte accumulation, stomatal regulation, and photosynthesis, leading to an improvement of plant growth and yield [49–52]. In terms of recognizing the most effective method to apply Zn applications, there has been some disagreement in the scientific community. Hussain et al. [53] demonstrated that the foliar method is a more appropriate and effective option than the soil method for attaining higher yield, which was in contrast with Ghoneim [54]. Differently, Arab et al. [55] indicated that in the case where the main goal was to achieve the highest yield, the choice of foliar applications of Zn was favorable, while to attain the highest silage, the soil application was favorable. Elshamly and Nassar [56] and Akbar et al. [57] found pronounced results by any sole application method of Zn (via soil and foliar) to improve plant growth and yield. While study of Shahab et al. [58] showed significant results by adopting combined applications.

Based on the aforementioned, it can be assumed that little data are available about the impact of the different applications of Kh and Zn under alternate or fixed partial root-zone drip irrigation techniques on the physiochemical traits, nutrient contents, yield, and water use efficiency of maize. Therefore, considering the above facts, this study hypothesized that Kh applications alone and in combination with Zn under alternate or fixed partial root-zone drip techniques could attain pronounced advantages in terms of water saving and maize plants.

## Materials and methods

### Experimental site description

The experiments were conducted during the 2021 and 2022 growing seasons at the experimental farm of Water Studies and Research Complex station, National Water Research Center, Southern of EGYPT, Abu Simbel City, located between (22° 24′ 11″ N and 31° 35′ 43″ E). Table 1 presents the soil properties (physical and chemical) of the location used in the field experiment. Soil physical and chemical properties were measured before the growing seasons 2021 and 2022. In each plot, at two interval depths of (0–30 and 30–60 cm) the soil samples were collected by using a spiral auger. Three soil samples were taken from each plot to make a composite sample. On the other hand, the distribution of soil particle size was determined by the hydrometer method, as described by Estefan et al. [59]. In accordance with USDA Soil Survey Staff [60] method, the soil texture was loamy sand. By following the standard procedures of Estefan et al. [59], other soil physical and chemical properties were measured. Whereas, soil pH and Electrical conductivity (EC) were measured before cultivating maize crops using a pH meter and an EC meter. The flame photometer method was used for the analysis of available potassium (K^+^) and sodium (Na^+^). Magnesium (Mg^2+^) was determined by titration method using EDTA, as outlined by Adams [61]. Organic matter contents in the soil samples were determined with the wet digestion method. The percent of CaCO_3_ was measured by using Collin’s calcimeter. Nitrogen (N) content was determined by using Kjeldahl method, while phosphorus (P) was measured by colorimeter methods using a UV–visible spectrophotometer at 410-nm wavelength. The available contents of iron (Fe) and Zn were determined by using an atomic absorption spectrophotometer.

**Table 1:** The (physical and chemical) properties of soil at the experimental site, Egypt before the growing seasons 2019/2020 and 2020/2021 (average of 2 years)

**Parameter**	**Unit**	Depth (cm)	
		0–30	30–60
**Mechanical analysis**			
Sand	(%) by weight	82.19 ± 0.21	81.1 ± 0.20
Silt	(%) by weight	13.16 ± 0.72	7.9 ± 0.11
Clay	(%) by weight	4.65 ± 0.70	11.0 ± 0.16
Texture	Loamy Sand		
**Chemical analysis**			
pH (paste extract)		7.9 ± 0.21	7.10 ± 0.31
Electrical conductivity (EC)	ds m^−1^	0.60 ± 0.01	0.60 ± 0.02
CaCO_3_	%	4.30 ± 0. 05	3.90 ± 0.07
Magnesium (Mg^+2^)	mg kg^−1^	2.40 ± 0.02	1.30 ± 0.07
Available Nitrogen (N)	mg kg^−1^	45.0 ± 0.70	46.0 ± 0.72
Available Phosphorus (P)	mg kg^−1^	8.9 ± 0.10	11.0 ± 0.15
Available Potassium (K)	mg kg^−1^	225.0 ± 0.70	205.1 ± 0.71
Available Iron (Fe)	mg kg^−1^	4.40 ± 0.10	4.9 ± 0.07
Available Zinc (Zn)	mg kg^−1^	0.95 ± 0.02	1.0 ± 0.02
Organic matter	(%) by weight	0.60 ± 2.0	0.2 ± 2.14

Each value represents the mean of the 3 replications ± standard errors

### Weather conditions

The area has a hyper-arid continental weather in summer and mild winter, with a mean temperature in the hottest month range between 18–34 °C, and scarce rainfall [62]. Table 2 presents monthly average values of relative humidity, temperature, precipitation, and wind speed during the 2021 and 2022 growing seasons. Throughout the growing seasons, the maximum temperature values were recorded in July during the first and second seasons, while the minimum values were recorded in November during the first and second seasons. Based on the results in Table 2, the temperatures recorded higher values in the first season than the second, in contrast to relative humidity values. Additionally, throughout the study periods; there weren't any precipitations.

**Table 2:** The weather data from the experimental site throughout the period (from July to November) during the growing seasons of 2019/2020 and 2020/2021

		**Temperature (°C)**		**Relative humidity (%)**		**Wind speed** **(Km hour**^**-1**^**)**	**Solar radiation (MJ m-**^2^**)**	**Precipitation (mm)**
		**Max**	**Min**	**Max**	**Min**			
July	2021	43.1±0.21	24.5±0.20	28.8±0.22	5.3± 0.25	2.5± 0.24	27.0± 0.20	0
	2022	42.7±0.20	24.3±0.21	28.1±0.21	3.3± 0.22	2.2± 0.24	26.2± 0.21	0
August	2021	42.3±0.21	25.4±0.19	31.3±0.22	5.8± 0.22	2.7± 0.22	20.5± 0.22	0
	2022	42.5±0.21	26.0±0.19	33.3±0.22	5.6± 0.22	2.6± 0.22	21.2± 0.21	0
September	2021	41.5±0.19	24.4±0.20	37.1±0.21	9.4± 0.21	3.2± 0.24	18.0± 0.19	0
	2022	40.7±0.20	26.7±0.21	37.5±0.22	9.3± 0.22	3.1± 0.21	18.6± 0.21	0
October	2021	36.5±0.20	21.0±0.21	43.0±0.21	12.6± 0.29	3.8±0.20	16.1± 0.18	0
	2022	36.2±0.20	21.3±0.21	48.2±0.23	16.1± 0.25	3.7±0.21	16.0± 0.20	0
November	2021	31.8±0.20	16.0±0.20	35.0±0.22	19.3± 0.25	2.5± 0.24	15.9± 0.21	0
	2022	31.2±0.21	15.8±0.20	36.3±0.19	20.2± 0.22	2.3±0.21	15.3± 0.22	0

Max maximum temperature, Min minimum temperature, and mm millimeter. The meteorological data were obtained from Toshka agrometeorological station, Egypt. Values are the mean of 3 replicates ± standard errors

### The experimental design and agronomic practices

The field experiment was conducted as a randomized split-split plot design with 3 replications under a drip irrigation system. In the main plots, two drip irrigation techniques included alternate partial root-zone drip irrigation (ADI, both halves of the maize plant zone alternately irrigated by one drip line) and fixed partial root-zone drip irrigation (FDI, only one fixed half of the plant sides irrigated by one drip line). Moreover, two irrigation water levels, i.e., 100 and 75% of irrigation water requirements (Ir) were allocated in the subplots. While the sub-sub plots were devoted to the nine applications, namely, control (pure tap water), Zn-F (chelated Zn sprayed on the maize leaves at the rate of 1.25 kg ha^−1^), Zn–S (chelated Zn applied through the drip irrigation systems at a rate of 1.25 kg ha^−1^ as soil applications), Kh-F (Kh applications sprayed on the maize leaves at a rate of 3 g L^−1^), Kh-S (Kh applications applied through the drip irrigation systems at a rate of 3 g L^−1^), Kh-F + Zn-F, Kh-F + Zn–S, Kh-S + Zn-F, and Kh-S + Zn–S. The applications of Kh were applied twice after 30 and 60 days from sowing. While, Kh applications purchased from Central China Trading Co. Ltd (KH; 75% humic acid + 4% fulvic acid + 2% Fe + 10% K_2_O) which were applied in two equal doses as recommended by the producing company. Applications of Zn in the form of chelated Zn EDTA (13%) were purchased from Loran Co., which were applied after 30 and 60 days from sowing.

On the 15 of July 2021 and 18 of July 2022, respectively, maize seeds (Giza 352- triple hybrid) were purchased from central administration for seed production, Egyptian Ministry of agriculture and land reclamation. Maize seeds were hand-sewn (two seeds/hill), 30 cm between hills within a row (drip line) and 75 cm between drip lines. The plot size was 5 m × 3 m having three drip lines of 4.5 m in length. The seeding rate of maize was 36 kg ha^−1^. After 14 days of cultivation, the plants were thinned to one plant. All plots were irrigated equally after sowing, and then after 22 days; the irrigation treatments were started. In the Ir_100_ irrigation level, maize plants were irrigated by applying full irrigation quantities (100% Ir) at all growth stages. While, in the Ir_75_ irrigation level, the plants were irrigated by applying 75% Ir, which was proportionally obtained from Ir_100_. The limited irrigation level (75%) was started from the development stage (22 days of sowing) till the harvest stage. Calcium superphosphate (15.5% P_2_O_5_) was applied at a rate of 710 kg ha^−1^, after 14 days of cultivation. Moreover, in 3 equal portions potassium sulfate (48% K_2_O) was applied at a rate of 235 kg ha^−1^ after 60, 75, and 90 days of sowing, respectively. Furthermore, ammonium nitrate (33.5% N) at a rate of 950 kg ha^−1^ was applied in equal doses (50 kg per dose), started after 14 days of sowing, and repeated every 3–4 days up to the flowering stage.

### Crop irrigation water requirements

A weather station was installed in the experiment site. The maize crop evapotranspiration (ETc) in the open field conditions was assessed through estimating reference evapotranspiration (ETo) by entering the metrological data in CROPWAT package, version 8.0 [63], this software package was marketed by FAO. Then, crop evapotranspiration (ETc) was calculated according to Allen et al. [64] as follows:

$$\text{ETc}=(\text{ETo }+\text{Kc})$$

**where**

ETc = crop evapotranspiration (mm day^−1^).

ETo = reference evapotranspiration (mm day^−1^).

Kc = single crop coefficient, dimensionless.

Maize crop growth stages (i.e. initial, development, mid, and late stage) were 22, 22, 45, and 18 days, respectively. While Kc values for Kc _ini_, Kc _mid_, and Kc _end_ stage were 0.24, 1.04, and 0.58, respectively according to Ouda [65]

The full irrigation water level amount (Ir_100_) was calculated according to the equation of Abd El-Wahed and Ali [66] as follows

$$\text{Ir }=\frac{\text{A}\times \text{ Etc}\times \text{Ii}\times \text{Kr}}{\text{Ea }\times 1000 \times \left(1-\text{Lf}\right)}$$

**where**

Ir = irrigation water requirements (mm).

A = plot area (m^2^).

Et_c_ = crop evapotranspiration (mm).

Ii = intervals between irrigation (day).

Kr = coverage coefficient (Kr = (0.10 + Gc) ≤ 1) to [67], Gc is ground cover.

Lf = leaching factor 10 % (since soil electrical conductivity is low, Lf was neglected).

Ea = irrigation system efficiency%, the efficiency for drip irrigation = 85%.

Then amount of Ir_75_ treatment was proportionally obtained from the Ir_100_. Accordingly, the applied irrigation water amounts during the growing seasons were (13,235 and 9926 m^3^ ha^−1^) for Ir_100_ and Ir_75_%, respectively.

### The measurements of Leaf water content (LWC)

The LWC contents were determined as described previously [68]. After 65 days of sowing in each plot, fully mature maize leaves were cut and collected from the randomly selected ten plants. Then, leaf fresh weight (LFW) of the samples were immediately recorded. The samples were immersed in distilled water and left in the dark at 4 °C overnight, blotted dry, and their turgid leaf weight (LTW) was recorded. The samples were then oven-dried at 70 °C for 24 h to determine the dry weight (LDW). the LWC was calculated from the equation

$$\text{LWC}= \frac{\text{LFW}-\text{LDW}}{\text{LTW }-\text{ LDW}}\times 100$$

where.

LFW: Leaf fresh weight.

LDW: Dry leaf weight.

LTW: Turgid leaf weight.

### Measuring the leaf hydrogen peroxide (H_2_O_2_) contents

The contents of H_2_O_2_ in maize leaves were determined according to Alexieva et al. (2001 [69]). To prepare the samples, 0.5 g of maize leaves were extracted by homogenizing at 4 °C with chilled trichloroacetic acid (TCA, 0.1%). Then, 0.5 mL aliquot of leaf extract was mixed well with 0.5 mL of phosphate buffer (100 mM, pH 7.0) and 2 mL of KI (1 M), and the mixture was incubated for one hour in the dark. After that, H_2_O_2_ content was measured in the absorbance by using a spectrophotometer at 390 nm and the standard curve of H_2_O_2_ and the results were expressed as μ mol g^−1^ FW.

### Assessing the antioxidant enzymes

The antioxidant enzymes contents were measured in the fresh maize leaves according to the method of Aebi [70] and Haider et al. [71] for CAT, Dhindsa et al. [72] for superoxide (SOD), and Senthilkumar et al. [73] for peroxidase (POD). Catalase (CAT) content was estimated by mixing one gram of fresh maize leaves with 4 mL of ice-cold extraction buffer. Then, 100 mL of the supernatant was added to 2.0 mL of the reaction mixture consisting of 100 mM phosphate buffer (pH 7.0) + 0.1 mM EDTA + 25 mM H_2_O_2_. The decrease in absorbance at 240 nm was monitored for one minute and computed to calculate the content of H_2_O_2_ decomposed. The SOD content was assayed in 1 g of fresh maize leaves by homogenizing the samples for five minutes with K-phosphate buffer pH 6.8 and 0.1 mm EDTA. The previous mixture was extracted, then (13 mm l-methionine + 75 µm nitro blue tetrazolium chloride (NBT) + 100 µm EDTA + 2 µm riboflavin in a 50 mm potassium phosphate buffer pH 7.8) was added. Finally, the absorbance was recorded at 560 nm, and the SOD contents were expressed in μ mol g^−1^ FW. To determine POD content in the fresh maize leaves, a reaction mixture consisting of 50 mL was homogenized with 3 mL phosphate buffer (0.1 M, pH 7.0) + 30 mL H_2_O_2_ (20 mM) + 50 mL guaiacol (20 mM). Then, the absorbance was recorded at 436 nm and the POD contents were expressed in μ mol g^−1^ FW.

### Total carbohydrate contents

After 65 days of sowing, the total carbohydrates were measured using a spectrophotometer at 630 nm. Briefly, 0.1 g fresh maize leaf tissue samples were homogenized in HCl (2.5 N), then the mixture was extracted. Total carbohydrate was measured by mixing one mL of the extract with 4 mL anthrone reagent, followed by boiling in a water bath at 100 °C for eight minutes then cooled to room temperature. Total carbohydrates were measured by using a standard curve made of glucose according to the method adopted by Hedge and Hofreiter [74].

### Proline content

Top maize leaves were randomly taken from ten plants after 65 days of sowing. The proline content was represented as μ mol g^−1^ FW and calculated according to Sahin [75]. For this purpose, the following processes were repeated three times, whereas 0.5 g of fresh leaf tissues were mashed on ice with 10 ml of 3% sulfosalicylic acid to homogenize After that, the resulting product was centrifuged and extracted. Then 2 ml of supernatant was extracted, and it was mixed with 3 ml of 2.5% ninhydrin reagent and 2 ml of glacial acetic acid. The mixture was placed in a 100 °C water bath, then immediately cooled on ice. After that 4 ml of toluene were mixed with the product. Finally, the solution was extracted, and by using a spectrophotometer set to detect absorbance at 520 nm.

#### Total chlorophyll content

The leaves' total chlorophyll content was determined by using a spectrophotometer according to Sadak et al. [76]. The maize leaves were collected from 10 random plants from each experimental unit. The total chlorophyll was determined in the 0.5 g of fresh maize leaf samples which were crushed and slowly mixed with 10 mL of acetone (80%). After that, the samples were left in the dark for 48 h then centrifuged at 10,000 g for five minutes. Then by spectrophotometer, the absorbance of the supernatant was read at 645 and 663 nm for chlorophyll a and b, respectively. The total chlorophyll content was measured in mg g^−1^ FW and calculated using the following formulas:

$$\text{Chlorophyll a}=12.7 (\text{A}663)-2.69 (\text{A}645)$$

$$\text{Chlorophyll b}=22.9 (\text{A}645)-4.68 (\text{A}663)$$

$$\text{Total chlorophyll }=\text{chlorophyll a }+\text{chlorophyll b}$$

#### Chemical analysis

At harvest, to determine mineral content in the seeds, oven-dried plant materials were first digested by nitric acid and H_2_O_2_ [59]. Then the concentration of nitrogen (N), Phosphorus (P), potassium (K), Magnesium (Mg), zinc (Zn), and iron (Fe) in-ground maize grains was estimated by using the atomic absorption spectrophotometer, as described by Abdelkader et al. [77] and Kumssa et al. [78].

#### Yield and yield components

At full maturity, ten maize plants were randomly taken from each plot to record the average of the following traits: the weight of ear, root length, 1000 grain index weight, and maize grain yield were determined for each plot and then converted to kg ha^−1^.

#### Irrigation water use efficiency (IWUE)

Mathematically IWUE was estimated according to Raza et al. [79] using the following formula:

$$\text{IWUE}= (\frac{\text{Y}}{\text{Ir }})$$

**where**

IWUE = irrigation water use efficiency (kg m^−3^).

Y = yield (kg ha^−1^).

Ir = applied irrigation water amounts (m^3^ ha^−1^).

#### Data analysis and statistics

Statistical analysis was performed by ANOVA and all the statistical analyses were run with Costat software program (version 6.303, 2004). Combined data of the two growing seasons were collected to analyze the individual and interactive effects. Irrigation (PRIs), irrigation levels, and examined applications were considered as fixed effects, while block was assigned as the random effect. The differences between mean values of PRIs, irrigation levels, examined applications, and their interactions were performed using the Tukey HSD post hoc tests at (p ≤ 0.05 level), as per Casella [80]. The present results are the means of three replicates ± standard deviation (SD).

## Results

### The impacts of the examined treatments on:

#### Nutrient uptake and soil pH

According to the results in Tables 3 and 4, the solitary effect of examined PRIs, irrigation levels, and examined applications (KH and Zn) affected N, P, K, Mg, Fe, Zn, and soil pH.

**Table 3:** The solitary effects of adopting water stress and applying different applications methods of potassium humate and zinc applications under partial root-zone drip irrigation techniques on maize nutrient uptake during the growing seasons of 2019/2020 and 2020/2021

Studied factors		N (g kg^−1^)	P (g kg^−1^)	K (g kg^−1^)	Mg (g kg^−1^)	Fe (mg kg^−1^)	Zn (mg kg^−1^)
**PRIs techniques**							
FDI		12.03 ± 4.2 b	1.31 ± 0.39 a	5.16 ± 1.4 a	0.949 ± 0.16 b	128.1 ± 21 b	35.3 ± 17.8 b
ADI		12.73 ± 3.9 a	1.22 ± 0.36 b	5.04 ± 1.5 b	1.07 ± 0.16 a	146.2 ± 20.6 a	43.5 ± 19.3 a
**Irrigation levels (Ir)**							
100 (%)		13.58 ± 2.8 a	1.28 ± 0.42 a	4.90 ± 1.2 b	1.011 ± 0.14 a	138.1 ± 21.7 a	39.2 ± 19.5 b
75 (%)		11.08 ± 3.6 b	1.26 ± 0.34 b	5.30 ± 1.5 a	1.012 ± 0.25 a	136.2 ± 33.4 b	39.6 ± 19.2 a
**Applications (EAP)**							
Control		9.85 ± 2.9 f	0.98 ± 0.13 e	4.15 ± 0.71 g	0.919 ± 0.17 h	118.4 ± 21.4 i	27.8 ± 7.8 i
KH F		12.24 ± 2.4 c	1.23 ± 0.24 c	4.65 ± 0.42 e	0.960 ± 0.17 f	126.1 ± 21.3 h	29.5 ± 7.9 h
Zn F		11.34 ± 1.6 e	1.16 ± 0.20 d	4.60 ± 0.34 f	0.943 ± 0.16 g	132.7 ± 21.6 g	33.4 ± 9.5 f
KH S		12.55 ± 2.5 bc	1.18 ± 0.19 d	5.18 ± 1.2 d	0.989 ± 0.18 e	138.1 ± 21.4 e	32.6 ± 4.9 g
Zn S		11.71 ± 2.6 d	1.13 ± 0.17 d	4.70 ± 0.74 e	0.983 ± 0.16 e	136.2 ± 21.5 f	42.2 ± 9.0 d
KH F + Zn F		11.59 ± 5.5 de	1.49 ± 0.4 a	5.61 ± 1.0 b	1.02 ± 0.14 d	142.3 ± 21.5 d	40.2 ± 7.5 e
KH F + Zn S		12.72 ± 4.3 b	1.38 ± 0.19 b	5.50 ± 0.77 c	1.06 ± 0.15 c	144.2 ± 21.6 c	48.9 ± 3.2 b
KH S + Zn F		14.69 ± 2.5 a	1.35 ± 0.21 b	5.58 ± 1.3 b	1.096 ± 0.14 b	147.7 ± 21.6 b	46.8 ± 6.9 c
KH S + Zn S		14.76 ± 1.2 a	1.47 ± 0.10 a	5.94 ± 1.1 a	1.12 ± 0.13 a	152.1 ± 21.6 a	53.6 ± 5.8 a
**Source of variation**	**df**	***p*-values**					
PRIs techniques	1	0.003	0.002	0.002	0.003	< 0.001	0.001
Irrigation levels (Ir)	1	0.001	0.009	< 0.001	0.348 NS	< 0.001	0.03
Examined applications (EAP)	8	< 0.001	< 0.001	< 0.001	< 0.001	< 0.001	< 0.001
PRIs × Ir	1	0.007	< 0.001	< 0.001	< 0.001	< 0.001	0.000
PRIs × EAP	8	< 0.001	0.004	< 0.001	0.589 NS	< 0.001	< 0.001
Ir × EAP	8	< 0.001	< 0.001	< 0.001	0.995 NS	< 0.001	< 0.001
PRIs × Ir × EAP	**64**	< 0.001	< 0.001	< 0.001	< 0.001	< 0.001	< 0.001

*Abbreviations*: *N* Nitrogen, *P* Phosphorus, *K* Potassium, *Mg* Magnesium, *Fe* Iron, *Zn* Zinc, *PRIs* Partial root-zone drip irrigation techniques, *FDI* Fixed partial root-zone drip irrigation, *ADI* Alternate partial root-zone drip irrigation. control (tap water applications), KH F (foliar applications of potassium humate = 3 g L^−1^ water), Zn F (foliar applications of chelated zinc = 1.25 kg ha^−1^), KH S (applications of potassium humate applied through the drip irrigation systems = 3 g L^−1^), Zn S (chelated zinc applied through the drip irrigation systems at a rate of 1.25 kg ha^−1^)

**Table 4:** The solitary effects of adopting water stress and applying different applications methods of potassium humate and zinc applications under partial root-zone drip irrigation techniques on soil pH, proline, hydrogen peroxide, carbohydrate, chlorophyll, and leaf water content in maize leaves during the growing seasons of 2019/2020 and 2020/2021

Studied factors		Soil pH	Proline (μmol g^−1^)	H_2_O_2_ (μmol g^−1^)	Carbohydrate (mg g^−1^)	Chlorophyll (mg g^−1^)	LWC (%)
**PRIs techniques**							
FDI		7.28 ± 0.1 a	108.7 ± 22 a	29.98 ± 11.3 a	526.3 ± 3.3 b	48.6 ± 12.4 b	72.37 ± 9.1 b
ADI		7.13 ± 0.08 b	102.3 ± 34 b	25.12 ± 11.9 b	621.4 ± 3.7 a	53.21 ± 11.0 a	77.48 ± 6.4 a
**Irrigation levels (Ir)**							
100 (%)		7.18 ± 0.08 b	97.4 ± 30.8 b	24.84 ± 12.2 b	539.5 ± 3.9 b	51.27 ± 10.6 a	77.09 ± 6.0 a
75 (%)		7.23 ± 0.2 a	113.6 ± 20 a	30.26 ± 10.5 a	608.2 ± 3.1 a	50.49 ± 13.9 b	72.76 ± 10.2 b
**Applications (EAP)**							
Control		7.27 ± 0.22 a	94.5 ± 18.4 h	23.26 ± 9.9 g	511.3 ± 1.9 h	44.2 ± 8.1 g	70.07 ± 10 h
KH F		7.233 ± 0.2 bc	96.2 ± 16.3 g	22.82 ± 6.8 g	532.8 ± 1.4 g	48.6 ± 4.5 e	72.47 ± 9.9 g
Zn F		7.23 ± 0.2 b	94.7 ± 20.6 h	21.05 ± 6.6 h	540.9 ± 0.52 f	47.8 ± 7.8 ef	72.22 ± 7.7 g
KH S		7.19 ± 0.15 d	96.9 ± 19.3 f	25.83 ± 10.1 f	570.9 ± 2.03 e	50.17 ± 5.0 d	74.89 ± 7.7 e
Zn S		7.22 ± 0.2 c	100.6 ± 19.4 e	27.98 ± 12.5 d	570.9 ± 0.35 e	47.2 ± 4.5 f	73.3 ± 7.8 f
KH F + Zn F		7.20 ± 0.23 d	106.0 ± 23.7 d	36.8 ± 5.9 a	633.5 ± 1.24 a	50.94 ± 6.06 d	75.69 ± 6.4 d
KH F + Zn S		7.18 ± 0.18 e	109.5 ± 28.6 c	33.23 ± 5.04 b	595.3 ± 0.52 d	53.46 ± 13.6 c	77.34 ± 5.3 c
KH S + Zn F		7.17 ± 0.15 e	114.6 ± 20.9 b	30.03 ± 8.4 c	606.3 ± 0.46 b	56.2 ± 9.5 b	78.45 ± 5.6 b
KH S + Zn S		7.14 ± 0.16 f	122.0 ± 37.6 a	26.95 ± 6.5 e	602.7 ± 0.32 c	59.3 ± 13.3 a	79.82 ± 4.5 a
**Source of variation**	**df**				***p*-values**		
PRIs techniques	1	0.001	< 0.001	< 0.001	< 0.001	< 0.001	< 0.001
Irrigation levels (Ir)	1	< 0.001	< 0.001	< 0.001	0.001	< 0.001	< 0.001
Examined applications (EAP)	8	< 0.001	< 0.001	< 0.004	< 0.001	< 0.001	< 0.001
PRIs × Ir	1	< 0.001	< 0.001	< 0.001	< 0.001	< 0.001	< 0.001
PRIs × EAP	8	< 0.001	< 0.001	< 0.001	< 0.001	< 0.001	< 0.001
Ir × EAP	8	< 0.001	< 0.001	< 0.001	< 0.001	< 0.001	< 0.001
PRIs × Ir × EAP	**64**	< 0.001	< 0.001	< 0.001	< 0.001	< 0.001	< 0.001

*Abbreviations*: *H*_*2*_*O*_*2*_ Hydrogen peroxide, *LWC* Leaf water content, *PRIs* Partial root-zone drip irrigation techniques, *FDI* Fixed partial root-zone drip irrigation, *ADI* Alternate partial root-zone drip irrigation. control (tap water applications), KH F (foliar applications of potassium humate = 3 g L^−1^ water), Zn F (foliar applications of chelated zinc = 1.25 kg ha^−1^), KH S (applications of potassium humate applied through the drip irrigation systems = 3 g L^−1^), Zn S (chelated zinc applied through the drip irrigation systems at a rate of 1.25 kg ha^−1^)

**Fig. 1:**
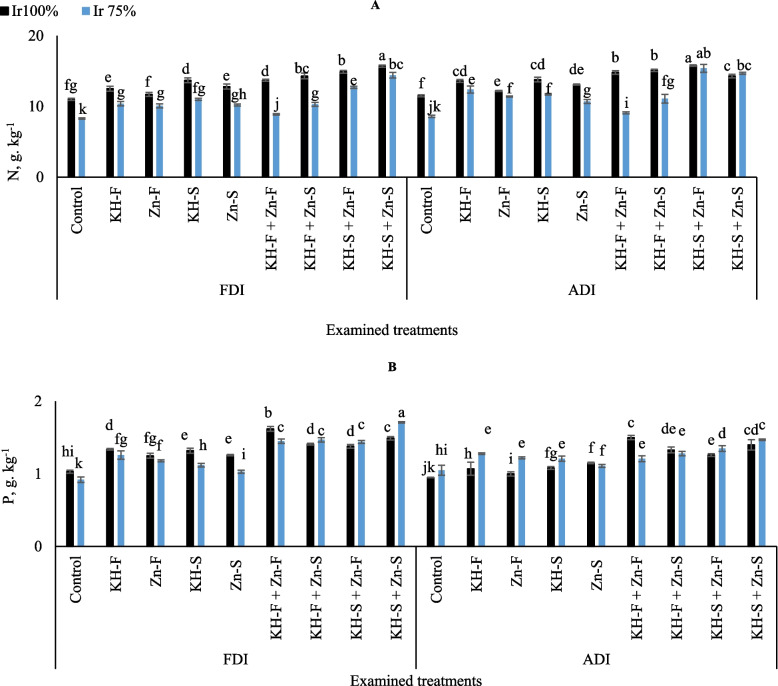
The interactive influences of the examined water levels and different applications methods of potassium humate and chelated zinc as individual or combined applications under fixed (FDI) or alternate (ADI) partial root-zone drip irrigation on **A**) nitrogen content (N) and **B**) phosphorus content (P) content. The results represent combined data of two growing seasons of 2021/2022. Error bars indicate standard errors from the mean. Bars with different letters are statistically significant at *p* ≤ 0.05

Generally, the obtained results in Fig. 1A showed that adopting stressful irrigation levels Ir_75_ in combination without or with applications of KH and Zn led to decreased N uptake contents. Interestingly, treating stressed maize plants with KH-S + Zn- F and KH-S + Zn- S applications under ADI led to equaled the same values for the well-irrigated plants. Hence, that could help maize plants to overcome the adverse impacts of water stress and improve N uptake contents. Overall, the highest N content was obtained by adopting Ir_100_ irrigation level under FDI and applying combined applications of KH-S + Zn- S; although that was significantly matched by adopting both irrigation levels under ADI and applying a combined application of KH-S + Zn- F. on the other hand, lowest values were recorded in control Ir_75_ under both examined PRIs.

The exposure of maize to the examined irrigation levels and different applications of KH and Zn significantly altered the levels of P in both examined PRIs (Fig. 1B). In the different treatments of KH and Zn under FDI, the adoption of Ir_75_ resulted in a notable decrease in P contents compared to Ir_100_ by 6.0, 15.2, 18.3, and 10.5% for KH-F, KH-S, Zn- S, and KH-F + Zn- F, respectively; and an increase by 5.0, 5.1, and 12.9% for KH-F + Zn- S, KH-S + Zn- F, and KH-S + Zn- S, respectively. Concomitantly, adopting ADI and exposure maize plants to Ir_75_ increased the levels of P by 16.4, 18.0, 10.7, and 6.7% for KH-F, Zn- F, KH-S, Zn- S, and KH-S + Zn- F respectively; and decreased by 19.3% for KH-F + Zn- F. The highest value was recorded under FDI by adopting the limited irrigation level and applying combined applications of KH-S + Zn- S and lowest value seen in control Ir_75_ under FDI and control Ir_100_ under ADI. However, in general, the interactive influences of the combined KH and Zn applications and irrigation levels showed better concentration in FDI as compared to ADI.

**Fig. 2:**
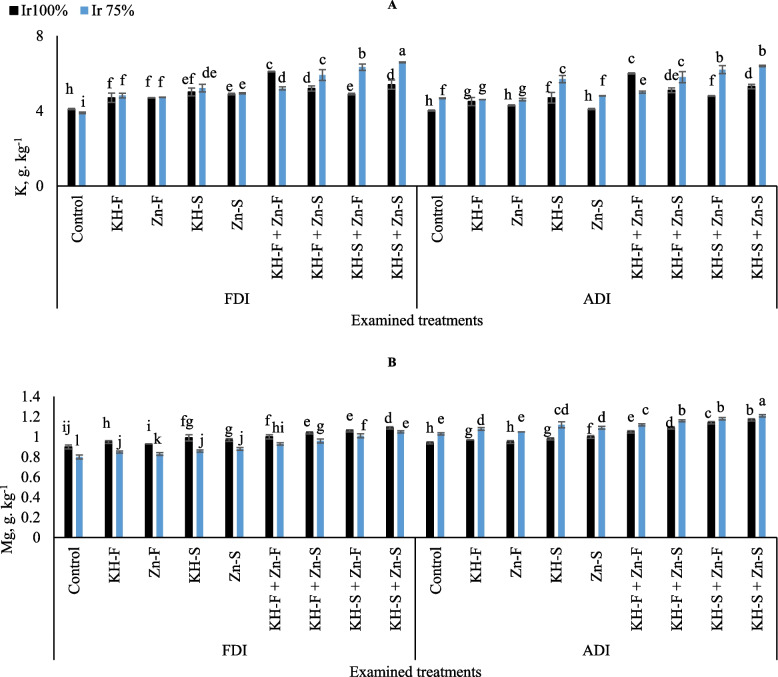
The interactive influences of the examined water levels and different applications methods of potassium humate and chelated zinc as individual or combined applications under fixed (FDI) or alternate (ADI) partial root-zone drip irrigation on** A**) potassium (K) and **B**) magnesium (Mg) content. The results represent combined data of two growing seasons of 2021/2022. Error bars indicate standard errors from the mean. Bars with different letters are statistically significant at *p* ≤ 0.05

The contents of K were improved by irrigating plants with limited irrigation level and applying the combined application of KH and Zn under both PRIs techniques, with an exception for KH-F + Zn- F (Fig. 2A) and this was pronounced in the plants exposed to Ir_75_ and examined fertilizer methods under FDI as compared to ADI. Relative to the control well-irrigated treatment under FDI, maize plants accumulated 32.8, 21.2, 16.3, and 24.1% higher K contents for KH-F + Zn- F, KH-F + Zn- S, KH-S + Zn- F, and KH-S + Zn- S, respectively. Likewise, relative to the control Ir_75_ treatment under FDI, maize plants accumulated 25.0, 34.0, 38.4, and 40.8% higher K contents for KH-F + Zn- F, KH-F + Zn- S, KH-S + Zn- F, and KH-S + Zn- S, respectively. Under ADI, relative to the control Ir_100_, the plants accumulated 33.0, 21.2, 16.2, and 24.2% higher K contents for KH-F + Zn- F, KH-F + Zn- S, KH-S + Zn- F, and KH-S + Zn- S, respectively. Similarly, relative to the control Ir_75_ treatment under ADI, maize plants accumulated 6.6, 19.5, 24.7, and 27.0% higher K contents for KH-F + Zn- F, KH-F + Zn- S, KH-S + Zn- F, and KH-S + Zn- S, respectively. Overall, the lowest K value was observed under FDI in the control Ir_75_ treatment. On the other hand, the obtained results showed that the adoption of applying the soil combined applications of KH-S + Zn- S to stressed maize plants attained the highest enhancement in K uptake under FDI irrigation technique.

In the different KH and Zn treatments, contents of Mg progressively augmented by adopting full irrigation requirement under ADI and decreased with the increment of drought stress under FDI irrigation technique, however, the increments of Mg contents in ADI were higher compared to FDI at all examined treatments (Fig. 2B). Generally, the highest Mg content was obtained by adopting Ir_75_ irrigation level under ADI and applying combined applications of KH-S + Zn- S. On the other hand, the lowest value was recorded in control Ir_75_ under FDI irrigation technique.

**Fig. 3:**
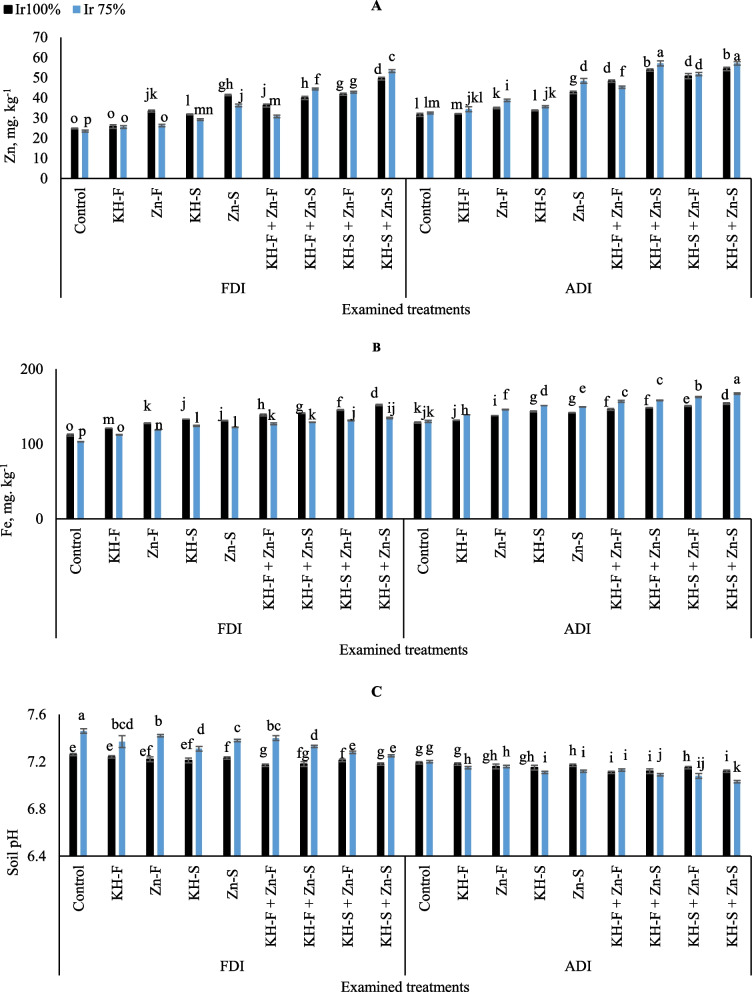
The interactive influences of the examined water levels and different applications methods of potassium humate and chelated zinc as individual or combined applications under fixed (FDI) or alternate (ADI) partial root-zone drip irrigation on** A**) zinc (Zn), **B**) iron content (Fe), and **C** soil pH. The results represent combined data of two growing seasons of 2021/2022. Error bars indicate standard errors from the mean. Bars with different letters are statistically significant at *p* ≤ 0.05

Illustrated data in Fig. 3A showed a substantial fluctuation in maize Zn uptake that was seen by adopting the different KH and Zn applications and using the examined irrigation levels under both PRIs techniques. There was a decrease in Zn contents in stressed irrigation level compared to Ir_100_ level under FDI treatments for Zn- F (21.3%), KH-S (8.2%), KH-F + Zn- F (12.3%) and there was an increase for KH-S + Zn- F (9.9%), KH-S + Zn- S (7.1%). Conversely, there were increases in Zn contents in most stressed treatments compared to full irrigation level under ADI treatments except for KH-F + Zn- F (6.2%). In general, the data showed that by adopting Ir_75_ under ADI irrigation technique, the highest Zn contents were observed by applying combined applications of KH-S + Zn- S, although that significantly equaled the adoption of Ir_75_ and applying combined application of KH-F + Zn- S under same irrigation technique. On the other hand, the lowest Zn content was observed in control Ir_75_ under FDI irrigation technique.

Exposure stressed maize plants to combined applications of KH and Zn significantly altered the levels of Fe in both PRIs techniques (Fig. 3B). There was a decrease in Fe contents compared to Ir_100_ level by 8.6, 8.6, 9.8, and 11.4% in FDI technique for KH-F + Zn- F, KH-F + Zn- S, KH-S + Zn- F, and KH-S + Zn- S, respectively. In contrast, Ir_75_ level and combined applications of KH and Zn had a positive effect and increased the Fe contents in the combined treatments by 6.9, 6.3, 7.4 and 7.8% under ADI technique. Overall, the highest Fe uptake was attained by adopting Ir_75_ irrigation level under ADI and applying combined applications of KH-S + Zn- S. While, the lowest Fe value was observed in control Ir_75_ under FDI irrigation technique.

To increase soil pH, it could be done by using limited irrigation level under FDI or using the solitary applications of KH and Zn in both PRIs techniques instead of the combined applications (Fig. 3C). However, that was pronounced in plants exposed to drought stress or treated with solitary applications under FDI as compared to the examined treatments in ADI. The lowest soil pH value recorded under ADI by adopting the limited irrigation level and applying combined applications of KH-S + Zn- S. While highest pH value seen in control Ir_75_ under FDI. However, in general he combined applications of KH and Zn alongside irrigation levels showed better effects under ADI technique as compared to FDI.

#### Proline, H_2_O_2_, carbohydrate, chlorophyll, and LWC

Based on the results in Table 4, the solitary effect of examined PRIs, irrigation levels, and examined applications (KH and Zn) affected proline, H_2_O_2_, carbohydrate, total chlorophyll, and LWC.

**Fig. 4:**
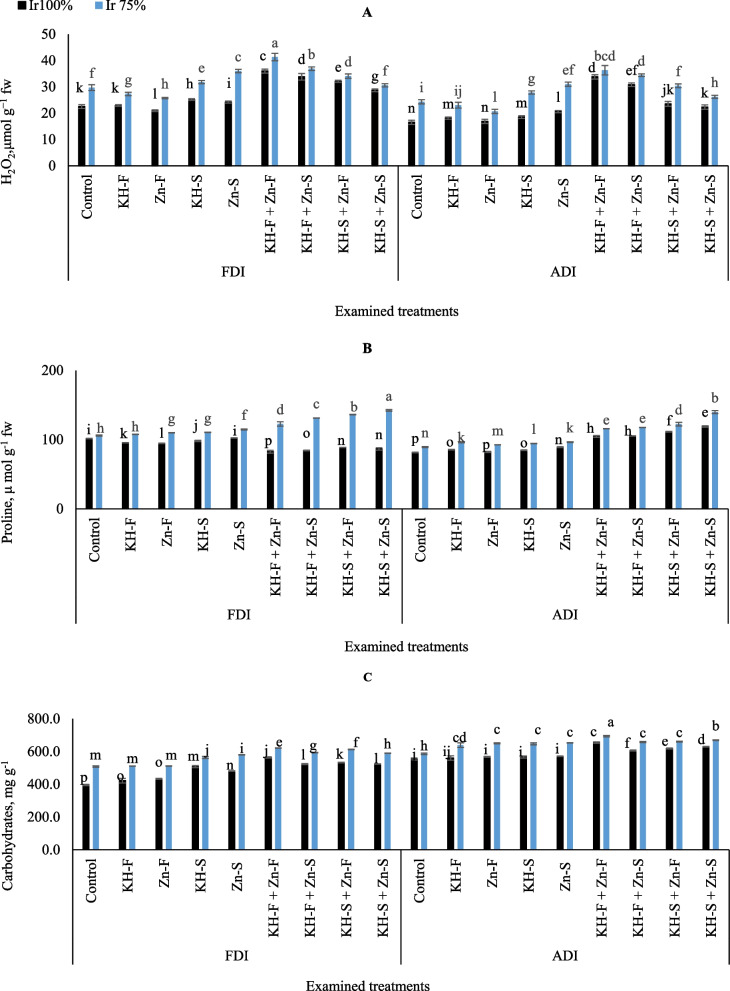
The interactive influences of the examined water levels and different applications methods of potassium humate and chelated zinc as individual or combined applications under fixed (FDI) or alternate (ADI) partial root-zone drip irrigation on **A**) hydrogen peroxide (H_2_O_2_), **B**) proline, **C**) and carbohydrate. The results represent combined data of two growing seasons of 2021/2022. Error bars indicate standard errors from the mean. Bars with different letters are statistically significant at *p* ≤ 0.05

The levels of H_2_O_2_ were increased by applying KH and Zn with the complete irrigation as well as with drought stress under both PRIs (Fig. 4A). However, this was pronounced in maize plants those exposed to drought stress and the combined applications under FDI as compared to unstressed plants under ADI. Based on the results, the highest H_2_O_2_ content was observed by applying the combined foliar applications of KH and Zn and adopting Ir_75_ under FDI technique.

The result in Fig. 4B presented the interactive influences of different applications of KH and Zn as in combination with examined water levels under PRIs. The results showed the ability of the examined water stress level and combined applications to produce proline and effectively maximize plant tolerance under FDI and ADI irrigation techniques. Regardless of treatments, generally, the interactive influences of KH and Zn applications in both irrigation levels under FDI had consistently higher levels of proline than ADI irrigation technique. Relative to the control well-irrigated treatment, maize plants accumulated 18.8, 16.9, 12.6, and 14.8% lower proline under FDI compared to 22.1, 22.7, 26.9, and 31.7% higher under ADI for KH-F + Zn- F, KH-F + Zn- S, KH-S + Zn- F, and KH-S + Zn- S, respectively. Likewise, relative to the control Ir_75_ treatment, maize plants accumulated 13.7, 19.3, 22.3, and 25.6% higher proline under FDI compared to 23.0, 24.2, 27.7, and 36.2% higher under ADI for KH-F + Zn- F, KH-F + Zn- S, KH-S + Zn- F, and KH-S + Zn- S, respectively. Overall, the lowest proline values were observed under ADI either in the plants without the examined fertilizers treatment and irrigated with full irrigation requirements, or in the plants treated with Zn- F at the same irrigation conditions. On the other hand, the obtained results showed that the adoption of supplying the KH-S + Zn- S to stressed maize plants attained the highest improvements in proline content under FDI irrigation technique.

Drought stress effectively increased the content of carbohydrates in maize leaves under two PRIs techniques and different application of KH and Zn compared to those of the well-irrigated plants (Fig. 4C). Generally, the lowest carbohydrate value was recorded in the plants under FDI without applied the examined applications and irrigated with full irrigation requirements. On the other hand, the obtained results showed that spraying the foliar applications of KH-F + Zn- F on stressed maize plants attained the highest improvements in carbohydrate content under ADI irrigation technique.

**Fig. 5:**
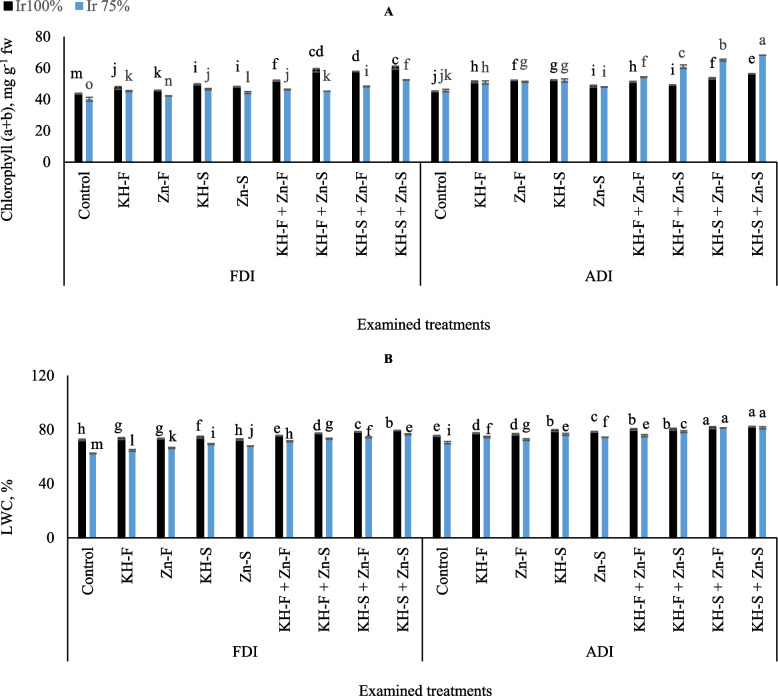
The interactive influences of the examined water levels and different applications methods of potassium humate and chelated zinc as individual or combined applications under fixed (FDI) or alternate (ADI) partial root-zone drip irrigation on **A**) total chlorophyll (a + b) and **B** leaf water content (LWC). The results represent combined data of two growing seasons of 2021/2022. Error bars indicate standard errors from the mean. Bars with different letters are statistically significant at *p* ≤ 0.05

The exposure of maize to the examined irrigation levels, PRIs, and different applications of KH and Zn significantly altered the levels of chlorophyll (a + b) (Fig. 5A). For instance, in the different applications of KH and Zn under FDI, adopting Ir_75_ level resulted in a notable decrease in chlorophyll (a + b) contents compared to Ir_100_. However, these decrements were pronounced in stressed maize plants exposed to the combined applications reached 10.8, 22.9, 16.4, and 13.1% for KH-F, KH-S, Zn- S, and KH-F + Zn- F, respectively. Concomitantly, under ADI notable increases in chlorophyll (a + b) contents were observed on stressed plants compared to the well-watered reached 5.5, 19.1, 18.0, and 17.3% for KH-F, Zn- F, KH-S, Zn- S, and KH-S + Zn- F respectively. The highest chlorophyll (a + b) value was recorded under ADI by adopting the limited irrigation level and applying combined applications of KH-S + Zn- S. On the other hand, the lowest chlorophyll (a + b) content was observed in control Ir_75_ under FDI.

Decreasing irrigation water requirements led to significant decrements in LWC percentage of maize under different examined applications and both PRIs, which equaled the obtained values in Ir_100_ treatments only by applying KH-S + Zn- F and KH-S + Zn- S under ADI irrigation technique (Fig. 5**B**). In general, the data showed that by adopting both examined irrigation levels under ADI irrigation technique, the highest LWC contents were observed by applying combined applications of KH-S + Zn- F and KH-S + Zn- S. On the other hand, the lowest LWC content was recorded in control Ir_75_ under FDI irrigation technique.

#### Antioxidant enzymes

According to the results in Table 5, the solitary effect of examined PRIs, irrigation levels, and examined applications (KH and Zn) affected antioxidant enzymes.

**Table 5:** The solitary effects of adopting water stress and applying different applications methods of potassium humate and zinc applications under partial root-zone drip irrigation techniques on antioxidant enzymes in maize leaves during the growing seasons of 2019/2020 and 2020/2021

Studied factors		SOD (μmol g^−1^)	POD (μmol g^−1^)	CAT (μmol g^−1^)
**PRIs techniques**				
FDI		243.4 ± 27.7 b	98.69 ± 27.9 b	328.5 ± 67.4 a
ADI		265.7 ± 61.5 a	122.62 ± 24.3 a	325.1 ± 61.1 a
**Irrigation levels (Ir)**				
100 (%)		242.5 ± 48.4 b	99.86 ± 31.2 b	309.5 ± 96.5 b
75 (%)		266.7 ± 45.02 a	121.5 ± 24.8 a	344.4 ± 69.5 a
**Applications (EAP)**				
Control		225.9 ± 52.5 g	100.7 ± 28.9 i	287.4 ± 62.9 e
KH F		241.3 ± 36.6 e	106.6 ± 33.2 g	305.7 ± 76.2 d
Zn F		238.9 ± 46.3 f	103.1 ± 31.5 h	301.1 ± 63.6 d
KH S		252.5 ± 37.6 d	110.2 ± 36.2 e	325.6 ± 77.2 c
Zn S		251.7 ± 27.8 d	108.1 ± 35.5 f	323.6 ± 70.9 c
KH F + Zn F		260.5 ± 29.6 c	112.0 ± 35.7 d	332.4 ± 74.9 c
KH F + Zn S		268.6 ± 33.3 b	115.9 ± 37.8 b	345.8 ± 83.9 b
KH S + Zn F		267.7 ± 51.1 b	115.1 ± 31.2 c	350.8 ± 101.3 b
KH S + Zn S		283.9 ± 53.1 a	124.2 ± 35.6 a	368.9 ± 99.9 a
**Source of variation**	**df**	***p*-values**		
PRIs techniques	1	< 0.001	< 0.001	0.19 NS
Irrigation levels (Ir)	1	< 0.001	< 0.001	< 0.001
Examined applications (EAP)	8	< 0.001	< 0.001	< 0.001
PRIs × Ir	1	< 0.001	< 0.001	0.47 NS
PRIs × EAP	8	< 0.001	< 0.001	< 0.001
Ir × EAP	8	< 0.001	< 0.001	< 0.001
PRIs × Ir × EAP	**64**	< 0.001	< 0.001	< 0.001

*Abbreviations*: *SOD* Superoxide dismutase, *POD* Peroxidase, *CAT* Catalase, *PRIs* Partial root-zone drip irrigation techniques, *FDI* Fixed partial root-zone drip irrigation, *ADI* Alternate partial root-zone drip irrigation. control (tap water applications), KH F (foliar applications of potassium humate = 3 g L^−1^ water), Zn F (foliar applications of chelated zinc = 1.25 kg ha^−1^), KH S (applications of potassium humate applied through the drip irrigation systems = 3 g L^−1^), Zn S (chelated zinc applied through the drip irrigation systems at a rate of 1.25 kg ha^−1^)

**Fig. 6:**
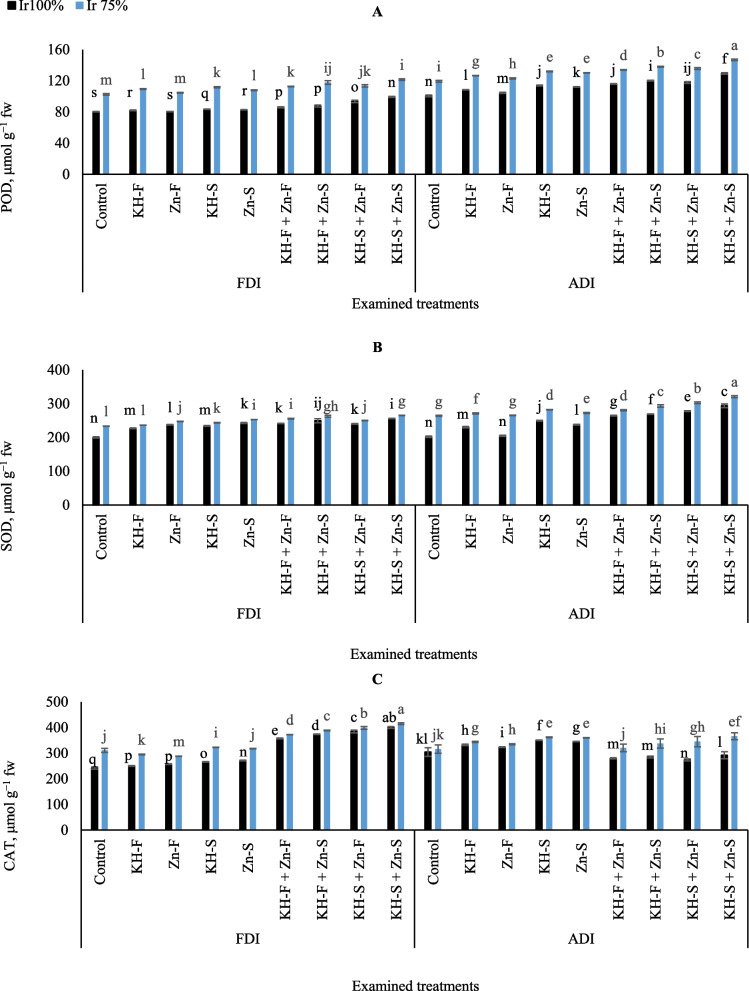
The interactive influences of the examined water levels and different applications methods of potassium humate and chelated zinc as individual or combined applications under fixed (FDI) or alternate (ADI) partial root-zone drip irrigation on **A**) Peroxidase (POD), **B**) Superoxide (SOD), and **C** Catalase (CAT). The results represent combined data of two growing seasons of 2021/2022. Error bars indicate standard errors from the mean. Bars with different letters are statistically significant at *p* ≤ 0.05

Decreasing irrigation water requirements led to a substantial rise in POD production in maize leaves under different examined applications and both PRIs (Fig. 6A). In general, the lowest POD values were observed under FDI either in the plants without the examined fertilizers treatment and irrigated with full irrigation requirements, and in the plants treated with Zn- F at the same irrigation conditions. On the other hand, the obtained results showed that the adoption of supplying the KH-S + Zn- S to stressed maize plants attained the highest improvements in POD content under ADI irrigation technique.

Similarly, with POD accumulations, a considerable enhancement in maize SOD accumulation was recorded after adopting the limited irrigation level or by using the examined KH and Zn applications under both PRIs irrigation techniques (Fig. 6B). However, the interactive impacts of the limited irrigation level either without the examined applications of KH and Zn or with their solitary applications under ADI had consistently higher enhancement of SOD than well-irrigated level under FDI irrigation technique. Whereas the results indicated that by comparing the examined applications under ADI, adopting Ir_75_ resulted in increased SOD contents compared to well-irrigated levels by 23.4, 15.0, 23.0, 11.5, and 12.8% for control, KH-F, Zn- F, KH-S, and Zn- S, respectively. Generally, the obtained results showed that the adoption of applying the combined soil applications of KH-S + Zn- S and adopting the limited irrigation level under ADI technique attained the highest improvements in SOD.

The result in (Fig. 6C) showed the ability of the examined irrigation levels and different applications of KH and Zn to increase CAT levels under FDI and ADI irrigation techniques compared to the control. However, generally, the interactive influences of the combined KH and Zn applications in both irrigation levels under FDI had consistently higher levels of CAT than ADI. While the interactive influences of the solitary KH and Zn applications in both irrigation levels under ADI had consistently higher levels than FDI. Relative to the control well-irrigated treatment under FDI, maize plants accumulated 32.5, 35.3, 36.9, and 39.5% higher CAT for KH-F + Zn- F, KH-F + Zn- S, KH-S + Zn- F, and KH-S + Zn- S, respectively. Likewise, relative to the control Ir_75_ treatment under FDI, maize plants accumulated 13.7, 19.3, 22.3, and 25.6% higher proline under FDI compared to 16.4, 20.0, 22.0, and 25.1% higher for KH-F + Zn- F, KH-F + Zn- S, KH-S + Zn- F, and KH-S + Zn- S, respectively. In general, under FDI supplying KH-S + Zn- S to stressed plants attained the highest CAT, although that was significantly equaled by adopting Ir_100_ under the same combined application and irrigation technique.

#### Maize agronomic traits

According to the results in Table 6, the solitary effect of examined PRIs, irrigation levels, and examined applications (KH and Zn) affected the agronomic traits of maize.

**Table 6:** The solitary effects of adopting water stress and applying different applications methods of potassium humate and zinc applications under partial root-zone drip irrigation techniques on maize agronomic traits during the growing seasons of 2019/2020 and 2020/2021

Studied factors		Root length (cm)	Ear weight (g plant^−1^)	Grain index (%)
**PRIs techniques**				
FDI		19.3 ± 5.0 b	201.6 ± 51.8 b	200.1 ± 59.9 b
ADI		24.3 ± 4.4 a	215.5 ± 64.8 a	211.5 ± 56.2 a
**Irrigation levels (Ir)**				
100 (%)		22.1 ± 4.4 a	206.7 ± 61.4 b	199.5 ± 39.4 b
75 (%)		21.6 ± 8.7 b	210.4 ± 58.9 a	212.1 ± 71.8 a
**Applications (EAP)**				
Control		19.2 ± 6.2 h	162.2 ± 24.7 e	160.5 ± 17.4 g
KH F		20.4 ± 5.9 f	181.6 ± 25.7 d	194.7 ± 41.8 e
Zn F		19.9 ± 5.9 g	179.0 ± 21.8 d	169.7 ± 20.5 f
KH S		21.3 ± 5.9 e	212.9 ± 23.5 c	221.2 ± 26 bc
Zn S		21.0 ± 5.6 e	213.5 ± 36 c	207.0 ± 21.5 d
KH F + Zn F		22.4 ± 6.1 d	216.7 ± 20.6 c	225.4 ± 21.4 b
KH F + Zn S		23.4 ± 6.1 c	231.5 ± 15.4 b	217.0 ± 31.8 c
KH S + Zn F		24.0 ± 6.13 b	233.6 ± 31.3 b	218.6 ± 51 bc
KH S + Zn S		25.0 ± 6.1 a	246.0 ± 42.8 a	238.4 ± 48.9 a
**Source of variation**	**df**	***p*-values**		
PRIs techniques	1	< 0.001	0.001	0.015
Irrigation levels (Ir)	1	< 0.001	0.012	< 0.001
Examined applications (EAP)	8	< 0.001	< 0.001	< 0.001
PRIs × Ir	1	< 0.001	0.002	0.127 NS
PRIs × EAP	8	0.997 NS	< 0.001	< 0.001
Ir × EAP	8	0.997 NS	< 0.001	< 0.001
PRIs × Ir × EAP	**64**	< 0.001	< 0.001	< 0.001

*Abbreviations*: *PRIs* Partial root-zone drip irrigation techniques, *FDI* Fixed partial root-zone drip irrigation, *ADI* Alternate partial root-zone drip irrigation. control (tap water applications), KH F (foliar applications of potassium humate = 3 g L^−1^ water), Zn F (foliar applications of chelated zinc = 1.25 kg ha^−1^), KH S (applications of potassium humate applied through the drip irrigation systems = 3 g L^−1^), Zn S (chelated zinc applied through the drip irrigation systems at a rate of 1.25 kg ha^−1^)

**Fig. 7:**
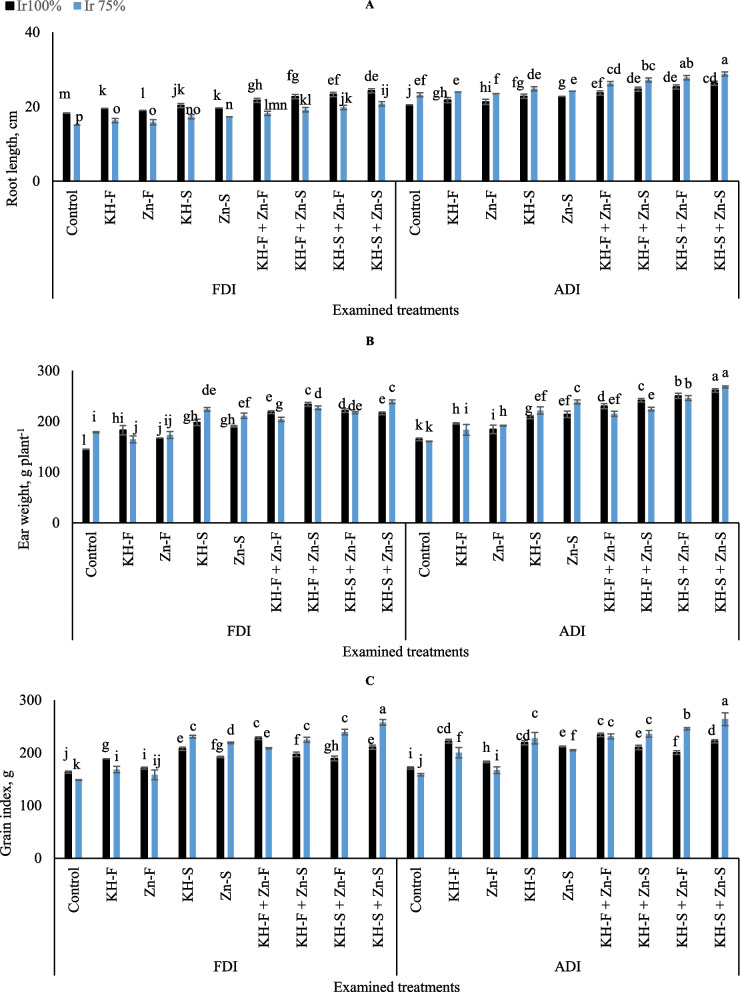
The interactive influences of the examined water levels and different applications methods of potassium humate and chelated zinc as individual or combined applications under fixed (FDI) or alternate (ADI) partial root-zone drip irrigation on **A**) root length, **B**) weight of ears, and **C** grain index weight. The results represent combined data of two growing seasons of 2021/2022. Error bars indicate standard errors from the mean. Bars with different letters are statistically significant at *p* ≤ 0.05

The exposure of maize to the examined irrigation levels, PRIs, and various applications of KH and Zn significantly changed maize root length (Fig. 7A). For instance, in the different applications of KH and Zn under FDI, adopting Ir_75_ level resulted in markable decreases in maize root length compared to Ir_100_. However, these decrements were pronounced in stressed maize plants exposed to the combined applications reached 16.1, 15.4, 15.0, and 14.4% for KH-F, KH-S, Zn- S, and KH-F + Zn- F, respectively. In contrast, under ADI notable increases in root length were observed on stressed plants compared to the well-watered reached 9.5, 9.2, 9.0, and 8.7% for KH-F, Zn- F, KH-S, Zn- S, and KH-S + Zn- F respectively. The highest root length was recorded under ADI by adopting Ir_75_ and either applying combined applications of KH-S + Zn- F or KH-S + Zn- S. On the other hand, the lowest root length was observed in control Ir_75_ under FDI.

The illustrated result in Fig. 7B showed a substantial fluctuation in the weight of maize ears which was seen by adopting the different KH and Zn applications and using the examined irrigation levels under both PRIs techniques. There was a significant increase in the ears weight in stressed irrigation level compared to Ir_100_ under FDI treatments for KH-S (11.6%), Zn- S (9.9%), KH- S + Zn- S (9.6%) and there was a decrease for KH-F (9.9%) and KH-F + Zn- F (6.4%). Under ADI treatments, there were significant increases in ear weight in stressed treatments compared to Ir_100_ for KH-S (6.0%), Zn- S (10.2%), and there was a decrease for KH-F + Zn- F (6.5%), and KH-F + Zn- S (7.2%). In general, the data showed that by adopting both irrigation levels under ADI irrigation technique, the highest ear weight of maize was observed by applying combined applications of KH-S + Zn- S. On the other hand, the lowest ear weight was observed in control Ir_100_ under FDI irrigation technique.

Grain index values were increased by applying KH and Zn under both PRIs and this was pronounced in the plants exposed to Ir_75_ compared to Ir_100_ in most treatments (Fig. 7C). Based on the results, the highest grain index values were observed by applying the combined soil applications of KH and Zn and adopting Ir_75_ under both examined PRIs. While the lowest grain index value was observed in control Ir_75_ under the FDI irrigation technique.

#### Yield and IWUE

Based on the results in Table 7, the solitary effect of examined PRIs, irrigation levels, and examined applications (KH and Zn) affected antioxidant enzymes maize grain yield and IWUE.

**Table 7:** The solitary effects of adopting water stress and applying different applications methods of potassium humate and zinc applications under partial root-zone drip irrigation techniques on maize yield and irrigation water use efficiencies during the growing seasons of 2019/2020 and 2020/2021

Studied factors		Yield (kg ha^−1^)	IWUE (kg m^−3^)
**PRIs techniques**			
FDI		4380 ± 2388 b	0.378 ± 0.2 b
ADI		6327 ± 2556 a	0.560 ± 0.3 a
**Irrigation levels (Ir)**			
100 (%)		5040 ± 2550 b	0.375 ± 0.1 b
75 (%)		5667 ± 3557 a	0.563 ± 0.3 a
**Applications (EAP)**			
Control		3911 ± 1552 h	0.345 ± 0.2 g
KH F		4945 ± 1670 f	0.428 ± 0.2 e
Zn F		4139 ± 1701 g	0.366 ± 0.3 f
KH S		4945 ± 1115 f	0.440 ± 0.3 e
Zn S		5221 ± 1713 e	0.457 ± 0.3 d
KH F + Zn F		6108 ± 1380 c	0.525 ± 0.2 c
KH F + Zn S		6350 ± 989 b	0.553 ± 0.2 b
KH S + Zn F		5902 ± 1444 d	0.522 ± 0.3 c
KH S + Zn S		6661 ± 876 a	0.588 ± 0.3 a
**Source of variation**	**df**	***p*-values**	
PRIs techniques	1	< 0.001	< 0.001
Irrigation levels (Ir)	1	< 0.001	< 0.001
Examined applications (EAP)	8	< 0.001	< 0.001
PRIs × Ir	1	< 0.001	< 0.001
PRIs × EAP	8	< 0.001	< 0.001
Ir × EAP	8	< 0.001	< 0.001
PRIs × Ir × EAP	**64**	< 0.001	< 0.001

*Abbreviations*: *IWUE* Irrigation water use efficiency, *PRIs* Partial root-zone drip irrigation techniques, *FDI* Fixed partial root-zone drip irrigation, *ADI* Alternate partial root-zone drip irrigation. control (tap water applications), KH F (foliar applications of potassium humate = 3 g L^−1^ water), Zn F (foliar applications of chelated zinc = 1.25 kg ha^−1^), KH S (applications of potassium humate applied through the drip irrigation systems = 3 g L^−1^), Zn S (chelated zinc applied through the drip irrigation systems at a rate of 1.25 kg ha^−1^)

**Fig. 8:**
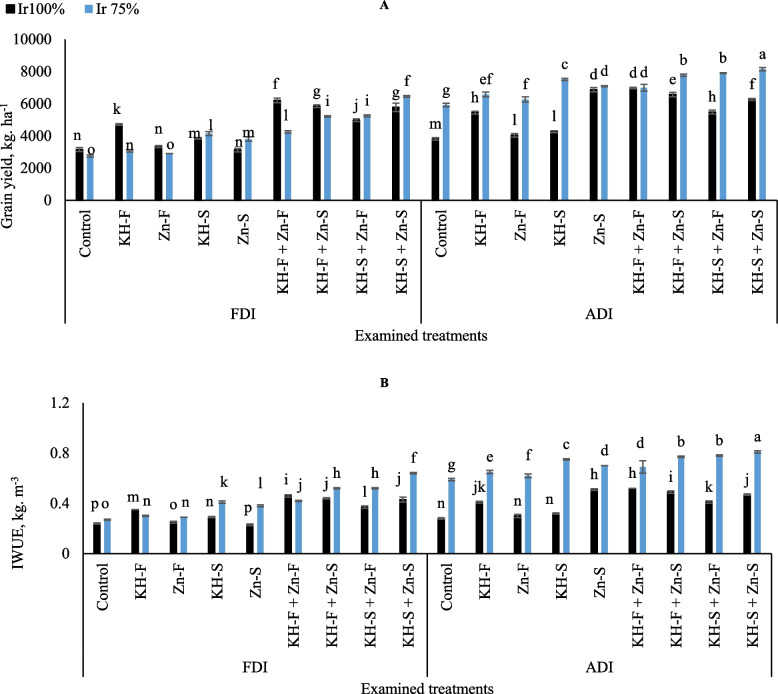
The interactive influences of the examined water levels and different applications methods of potassium humate and chelated zinc as individual or combined applications under fixed (FDI) or alternate (ADI) partial root-zone drip irrigation on **A**) grain yield and **B** irrigation water use efficiency (IWUE). The results represent combined data of two growing seasons of 2021/2022. Error bars indicate standard errors from the mean. Bars with different letters are statistically significant at *p* ≤ 0.05

Relative to the control well-irrigated treatment under FDI, maize plants produced 49.1, 46.0, 36.5, and 45.4% higher grains yield for KH-F + Zn- F, KH-F + Zn- S, KH-S + Zn- F, and KH-S + Zn- S, respectively (Fig. 8A). Likewise, relative to the control Ir_75_ treatment under FDI, maize produced 35.2, 47.2, 47.5, and 57.4% higher grain yield for KH-F + Zn- F, KH-F + Zn- S, KH-S + Zn- F, and KH-S + Zn- S, respectively. Under ADI, relative to the control Ir_100_, the plants produced 45.3, 41.9, 30.4, and 39.1% higher yields for KH-F + Zn- F, KH-F + Zn- S, KH-S + Zn- F, and KH-S + Zn- S, respectively. Similarly, relative to the control Ir_75_ treatment under ADI, maize plants produced 15.4, 23.8, 25.1, and 27.3% higher maize yields for KH-F + Zn- F, KH-F + Zn- S, KH-S + Zn- F, and KH-S + Zn- S, respectively. Overall, the lowest grain yield was observed under FDI in control Ir_75_ treatment, although that was significantly equaled by applying Zn- F under the same irrigation level and irrigation technique. On the other hand, the obtained results showed that the adoption of applying the soil combined applications of KH-S + Zn- S to stressed maize plants attained the highest grain yield under ADI irrigation technique.

Similar to maize grain yield, the illustrated results in Fig. 8B showed a substantial fluctuation in IWUE of maize, which was observed by adopting the various KH and Zn applications and using the examined irrigation levels under both PRIs techniques. Generally, the interactive influences of the combined KH and Zn applications were more pronounced in the limited irrigation level under ADI. Based on the obtained results, by adopting a limited irrigation level under ADI irrigation technique, the highest IWUE of maize was observed by applying combined applications of KH-S + Zn- S. On the other hand, the lowest IWUE was observed in control Ir_75_ under FDI irrigation technique.

## Discussion

In arid regions, appropriate strategies of irrigation and fertigation are required to improve nutrient uptake, plant development, yield, and water use [81]. From this perspective, this study sought to identify the best fertigation and irrigation management under full and drought stress conditions.

### Nutrient status under irrigation techniques, irrigation levels, and examined fertilizer methods

The results revealed significant decreases in the tested nutrients, including N, P, Mg, Zn, and Fe in response to the tested level of drought stress and sole examined fertilizers under FDI. While the drought-induced repression under ADI increased (P, K, and Mg) and decreased N uptake.

Such results under FDI are mainly attributed to the drought-induced restriction in root system (sizing root development), which decreases root activities within drip moisture range. In addition, during prolonged drought stress, accumulations of alkaline salt in topsoil layers increase, leading to increased soil pH and N loss by nitrification [82, 83]. Hence, under such conditions; the availability and uptake of (N, P, Mg, Zn, and Fe) decreased [84, 85]. These findings are consistent with the reported reductions in the N and P uptake under prolonged deficit irrigation [86–88]. Also, consistent with that, it has been reported that droughted plants face a severe deficiency of nutrient elements as a consequence of drought stress [89].

On the other hand, by adopting Ir_75_ irrigation level and most examined fertilizers of KH and Zn under ADI irrigation technique, the results showed positive impacts on P, K, and Mg uptake. These findings highlight the dual potential of irrigation techniques and appropriate irrigation levels as effective strategies to boost nutrient uptake status and plant development. In the study, the limited water applications under ADI significantly improved root length and diffusion within the soil rhizosphere when irrigation water was applied in rotation wet/dry cycles. However, the plants' have to deal with prolonged drought stress. Additionally, limited irrigation water led to generate an auxiliary source of H^+^ production and increase acidic secretions in soil layers (lower pH values), which could easily result in improving the uptake of P, K, and Mg [90]. These findings are thus in harmony with Bouray et al. [91], who reported that drought-stressed plants prioritize alteration to root architecture, resulting in different morphological and chemical changes such as (e.g., root length, root density, extracellular enzyme productions, and organic anions secretion), allowing plants to increase nutrients acquisition.

On the other side, decreasing irrigation can promote the reduction in N uptake in arid and semi-arid regions [92], whereas insufficient irrigation and higher temperature degrees under these conditions may increase oxidation processes, affecting the availability of N, and ultimately impacting N utilization [93, 94]. In this respect, these findings are also in harmony with Kirschbaum [95], who reported that every 1 °C increase in soil temperature, resulting in a loss in soil organic carbon and nitrogen could reach up to 10%. Similarly, Liu et al. [96], found that limited water level can lead to a decline in the N utilization ability of plants, which is related to the low soil N availability and reductions in root uptake capacity toward N under these conditions. Therefore, these results summarize the potential reasons of these reductions in N uptake by adopting limited irrigation levels under ADI irrigation technique.

By providing stressed maize plants with combined applications of KH and Zn would help plants overcome the adverse impacts of drought stress on the nutrient uptake status. The current results revealed significant positive impacts in the nutrient uptake, including P, K, and Zn in response to the tested level of drought stress and different combined applications of KH and Zn, with an exception of KH -F & Zn- F under both irrigation techniques. Such findings are mainly attributed to the returned benefits of the soil applications compared to the foliar applications of KH and Zn, which increase P, K, and Zn uptake under the same growth conditions. Consistent with that, it has been reported that drought-induced perturbation in nutrient availability especially those affected by higher pH values such as P and Zn [18, 43, 56]. Hence, supplying stressed maize plants with KH and Zn as soil applications would allow them to decrease soil pH as consequence of increasing plant exudates and prevent nutrient fixation. The increased availability in P and Zn leads to an increase in (P, K, and Zn) uptake, thereby decreasing water potential and enhancing the water status of the plant, these findings are in agreement with [97, 98]. Moreover, under drought stress conditions plants tend to decrease their activities including the transpiration process by closing stomata to maintain water within plant tissues. The closure of stomata leads to reductions in nutrient pathways to getting through the leaves, which limits foliar fertilization efficiency and nutrient uptake. Furthermore, Chtouki et al. [99] reported that under mild drought level or P-insufficient conditions, higher ratios of root/shoot were observed, which enhanced the absorption of nutrient and water. Under similar condition roots play the vital role in controlling plant activities [100]. Therefore, applied KH and Zn as foliar applications decreased P, K, and Zn uptake, which consistent with previous finding of Zanin et al. [101].

### Physio-biochemical responses under irrigation techniques, irrigation levels, and examined fertilizer methods

Under drought conditions and both irrigation techniques, maize plants undergo different mechanisms to deal with the reduction in water irrigation. Among these mechanisms are proline, antioxidant enzymes, and total carbohydrates. These results are consistent with the reported accumulations of these components in field-grown maize [43], and in field-grown sorghum [102] under limited water availability. However, a remarkable accumulation of antioxidant enzymes was observed under ADI (Ir_75_), potentially due to its effective advantages in forming a strong root and defense system compared to FDI (Ir_75_). Whereas, in the present study under ADI, drought stress induces the generation of a series of morphological and biochemical impacts resulting in increment of root elongation, root secretions, and augmented activity of nutrient acquisition [21, 103, 104]. By increasing water and nutrient uptake and enhancing plant growth, maize plants aimed to optimize the defense system and increase the synthetic process. These adaptive responses helped maize plants maintain physio-biochemical functions and improved plant resilience to drought stress.

The study results revealed a significant reduction in the chlorophyll (a + b) and LWC content in response to the tested level of drought stress and tested fertilizers under FDI. However, decreasing irrigation water under ADI irrigation technique significantly improved chlorophyll (a + b) and LWC content compared to Ir_100_ irrigation level. The significantly higher chlorophyll content markers in ADI, compared to FDI under drought stress conditions, suggest that ADI irrigation technique might have improved (Mg, Zn, and Fe) uptake, those involved in chlorophyll synthesis. In this respect, the role of Mg and Fe in chlorophyll synthesis is well known as reported in previous studies [105–107]. Moreover, the stimulatory effect of Zn on chlorophyll synthesis may be due to its positive contribution to the structural components of various proteins, enzymes, and co-factors [49, 108].

Based on the aforementioned findings, reductions in the (chlorophyll and LWC) and enhancements in osmolytes and H_2_O_2_ are critical consequences of drought stress. In this investigation, water and nutrient limitation substantially declined in response to the tested level of drought, with more pronounced adverse effects under FDI than ADI irrigation technique. Hence, the current study has hypothesized that supplying maize plants with KH & Zn would allow them to cope with the reduction in the water irrigation amounts and also improve plant water status and defensive mechanisms.

Accordingly, the findings indicated that various KH and Zn applications especially under ADI accumulated significant high levels of antioxidant enzymes, carbohydrates, chlorophyll (a + b) and low significant differences in LWC in response to increased water limitation. These findings could be attributed to the utilization of KH and Zn resulting in the enhancing levels of plant nutrient status, and the benefits of ADI might also have a stimulatory effect on further nutrient solubilization.

It is worth noting that the pronounced improvements in proline content especially under FDI were unpredicted, particularly with the observed reductions in N uptake under drought stress conditions, which disagrees with the reported concept as N plays a crucial role in the proline synthesis. These results indicate that the stressed maize plants specify and change their main physiological and biochemical processes according to the status quo and progress by providing consumption in the energy units during stress conditions. For clarification, the adoption of FDI (Ir_75_) progressively catalyzed (LWC, N, Mg, Zn, and Fe) deficiency. To alleviate drought-induced cellular water and nutrient deficiency, and to sustain water osmotic homeostasis, plants rely upon physiological responses such as osmotic adjustment, nutrients selection, and antioxidant systems activation. The results indicated that maize accumulated significant higher levels of P and K compared to ADI in response to applied combined applications of KH & Zn under FDI and increased water limitation up to 75%. Additionally, the reduction in nutrient uptake (N, Mg, Zn, and Fe) seems forced maize plants to another selection in synthesis processes. Under these conditions, plants decreased chlorophyll synthesis and redirected the most absorbed N amounts to accelerate proline and CAT synthesis. Hence, proline production continued, causing substantial reductions in water potential. These reductions resulted in some enhancements in water absorption and water status under these conditions. Similarly, Kapoor et al. [109] reported that proline is a key component in adjusting plants' osmotic pressure, hence proline concentration significantly rises with an increase in water stress. At the same time, the plants tend to increase carbohydrate synthesis, by increasing P uptake. Hence, the above plant strategies including high uptake levels of P, K, carbohydrate accumulations, proline accumulations, and the selection of certain physiological pathways contribute significantly to the regulation of osmotic adjustment, nutrient selection, activation of scavenging excess ROS; responses that ultimately improve water absorption, nutrient uptake, and plant resistance. Also, increased production of CAT under these conditions protects maize cells from rising levels of ROS species, boosting the antioxidant capacity and enhancing maize's ability to resist water deficit; these findings agree with Hasanuzzaman et al. [110]. In this respect, Alam et al. [111] highlighted that CAT is the most efficient antioxidant than others to neutralize H_2_O_2_ into H_2_O and O_2_. Moreover, studies by Alam and Ghosh [112] and Poli et al. [113] have noticed a higher level of CAT antioxidant compared to the other antioxidants to resist ROS species at the early plant stages. This highlights the importance of studying maize behaviors and responses under water deficit to find a suitable technique that allow plants to adapt in unfavorable conditions.

On the other hand, the study found that using ADI irrigation technique and most different dosages of KH & Zn applications did not change the adoption of the previous strategies. However, applying KH-S & Zn- F and KH-S & Zn- S during Ir_75_ irrigation level significantly changed the obtained strategies under the ADI irrigation technique. Whereas, the study found that these applications significantly changed the plants nutritional and defensive mechanisms. For instance, the applications of KH-S & Zn- F and KH-S & Zn- S helped maize plants to moderate the effect of drought stress on the N uptake. Consequently, it seems that these applications improved proline and chlorophyll synthesis which contribute to the drought tolerance under ADI. Also, it was observed that drought stress under ADI technique resulted in an improve maize root lengthen and root acquisition, and this might be due to drying and rewetting circles which could make a wide change in the nutrient availability, making it more susceptible to root activity. Moreover, the presence of permanent stress degrees owing to the wet/dry circles has positively impact on proline contents, however, these contents were less compared to those recorded under FDI (Ir_75_). Furthermore, the simultaneous application of (KH-S & Zn- F and KH-S & Zn- S) decreased soil pH, and increased nutrient availability, and nutrient uptake. This resulted in higher levels of absorbed (P, K, Mg, and Fe) in maize, leading to enhancements in physiological processes such as photosynthesis and LWC, ultimately enhancing plants' stress tolerance. Similar results have been reported in previous studies by [114–117].

These modifications show maize plants' dynamic responses to KH & Zn applications under water deficit conditions and highlight the fundamental role of PRIs technique in reregulating the tolerance processes that allow maize plants to survive in these diverse circumstances.

### Yield, WUE, and IWUE under irrigation techniques, irrigation levels, and examined fertilizer methods

Each of crop yield, WUE, and IWUE are influenced by the interaction relationships between irrigation water and appropriate fertilization. Compared with FDI irrigation technique, maize grain yield, straw yield, WUE, and IWUE attained the maximum improvement in limited irrigation level and an appropriate soil combination of Kh-S & Zn- S under ADI. Enhanced yield, WUE, and IWUE in Kh-S & Zn- S-treated plants under ADI might be due to increased P, K, and Mg uptake, which leads to enhanced water status, root efficiency, and carbohydrate biosynthesis and transport [41, 118]. Moreover, limited irrigation level may improve the roots' length and effectiveness, resulting in an enhancement in maize development. Under these circumstances, the external application of KH-S & Zn- S caused a significant increase in osmotic adjustment [119], nutrient metabolism and photosynthesis process [120, 121], plant cell membrane stability, chlorophyll formation, and osmolyte accumulation [49, 51, 52]. These influences led to enhanced absorption of water and nutrients, which ultimately increased the maize plants' tolerance to drought stress and improved yield, WUE, and IWUE.

## Conclusion

In the presence of deficit irrigation, combined applications of potassium humate and chelated zinc resulted in significant improvements in the contents of P, K, proline, and CAT under fixed partial root-zone irrigation. This strategy which represents of higher accumulations of proline and CAT in maize plants under fixed partial root-zone irrigation technique might play a potential role to reduce the oxidative damage induced by drought stress. Interestingly, under alternate partial root-zone drip technique the previous strategy was modified and the adverse effects of water stress were mitigated by the combined applications of potassium humate and chelated zinc as a soil application. Contents of Mg, Zn, Fe, POD, SOD, chlorophyll (a + b), and leaf water content were increased under alternate partial root-zone drip technique in plants those treated with soil combined applications of potassium humate and chelated zinc which may have a significant role for enhancing yield and water use efficiency of maize under water deficit condition. The current investigation concluded that these soil applications in association with alternate partial root-zone drip technique can be used as a promising technology in the agroecosystems to ameliorate deficit water stress effects on maize growth development.

## Data Availability

The data used or analyzed in this study are included in this article. Other materials that support the findings of this article are available from the corresponding author on reasonable request.
